# Residual Stress Distribution Monitoring and Rehabilitation in Ferromagnetic Steel Rods

**DOI:** 10.3390/s22041491

**Published:** 2022-02-15

**Authors:** Kaiming Liang, Spyridon Angelopoulos, Aphrodite Ktena, Xiaofang Bi, Evangelos Hristoforou

**Affiliations:** 1 Key Laboratory of Aerospace Materials and Performance (Ministry of Education), School of Materials Science and Engineering, Beihang University (BUAA), Beijing 100191, China; liangkm@buaa.edu.cn; 2 Laboratory of Electronic Sensors, Zografou Campus, National Technical University of Athens, 15780 Athens, Greece; spyrosag@central.ntua.gr; 3 Energy Systems Laboratory, Evripos Campus, National & Kapodistrian University of Athens, 15780 Athens, Greece; apktena@uoa.gr

**Keywords:** residual stress measurement, magnetic rod, stress annihilation, localized induction heating

## Abstract

Different means of residual stress distribution monitoring in magnetic rods are illustrated in this paper, through measurements of permeability, magnetoelastic uniformity using two different setups, sound velocity, and eddy currents. The effectiveness of these techniques was assessed through the stress monitoring of the same magnetic rod, suffering residual stresses in two known volumes caused by controlled hammering. Furthermore, rehabilitation has been achieved by means of stress annihilation, achieved by localized induction heating. As a result, the magnetoelastic and sound velocity uniformity measurements are more appropriate for the monitoring of localized residual stresses, while eddy current measurements are useful for the monitoring of the geometrical deformation.

## 1. Introduction

Residual stress distribution monitoring and rehabilitation in steels is one of the most important key performance indicators (KPI) in steel industry [1]. The ability to perform so allows for faultless production, manufacturing, and certification of steels and related products, thus offering a disruptive advantage to those industries applying such a technology [2].

The stress gradient along the length of a steel product is responsible for the generation of dislocations and dislocation forests, resulting in the generation of nano-cracks [3]. Thus, stress distribution monitoring with respect to time can predict the generation of dislocations and nano-cracks. Furthermore, the ability to monitor the three-dimensional residual stress distribution—i.e., the monitoring of the stress tensor—permits the prediction of nano-cracks in three dimensions. It goes without saying that stress rehabilitation—i.e., processes that restore stress levels to desired levels—offers a disruptive tool to the steel industry that employs this technology.

However, residual stress distribution monitoring is not a trivial sensing method. At the moment, the two basic technologies for stress measurement and monitoring are the strain gauge [4] and the hole drill [5,6] techniques. The strain gauge method is based on the change of the resistivity of a metallic sensing element, glued (or connected by any non-destructive means) on the tested surface (in the surface of the tested steel for the needs of the current study). Such a technique offers the measurement of tensile and compressive stresses in the volume beneath the strain gauge, after the strain gauge is firmly set on top of the tested surface. The strain gauges may also offer the ability of monitoring the stress vector by setting them in different orientations. The hole drill method is based on the generation of a hole, either nano-hole or micro-hole, on the tested surface. The pre-existing stresses cause a deformation of the circular hole to an ellipse, showing the history of residual stresses at the volume around the hole, which is a certain advantage with respect to the strain gauge method, despite the fact that the hole drill method is a destructive method in principle, although surface nano-hole generation may be considered as a least destructive approach. The orientation of the ellipse demonstrates the direction of the local stresses, while the ratio between the large and the small diameter offers the amplitude of the stress vector. Monitoring the change of the ellipse in time permits the monitoring of the stress tensor at the point, where the hole has been generated. Thus, both methods are able to monitor the stress tensor at the point, they have been set or generated. By definition, it is impossible to monitor stress distribution, as they are fixedly set at a given surface area of the tested steel.

The standard laboratory methods of monitoring residual stresses on surfaces and in the bulk of materials, including steels, are the X-ray diffraction in the Bragg–Brentano set-up, XRD-BB in short [7], and the neutron diffraction, ND in short [8], respectively. Following these two methods, the residual stress tensor distribution of a given steel coupon can be realized, either for the surface (XRD-BB) or the bulk (ND) of the coupon. Both of these methods offer an uncertainty in stress monitoring in the order of 5%, while recent advances permitted improving the uncertainty down to 1%. The use of these techniques is permissible only in lab scale for obvious reasons. However, these two methods have been used as a reference calibration for other techniques, which may be applicable in the field and employ non-linear magnetic and acoustic methods. Magnetic methods monitor surface or bulk magnetic parameters using single sheet testers or electromagnetic yokes. The most commonly used methods are those monitoring changes in the magnetic permeability, coercivity, or anisotropy [9,10,11] and the magnetic Barkhausen noise [12,13,14,15,16,17]. Other magnetic methods include the magnetic memory method [18] which permits the qualitative assessment of changes in stress distribution or the magnetic adaptive testing method, relying on the processing of a considerable amount of minor loop data [19]. The well-established correlation of the magnetic Barkhausen noise (ΜBN) with stress has led to a variety of laboratory setups and commercial equipment which perform very well at laboratory scale measurements but suffer large geometrical uncertainties in the field. Several metrics and processing methods, including modeling, have been proposed to extract actionable information from MBN data concerning stresses in the elastic and plastic region [16,20,21]. The acoustic methods proposed are based on the non-linear amplitude of the propagating elastic waves or the non-constant longitudinal-transverse sound velocity of the metal (steel in our case) [22,23]. Eddy current techniques have also been proposed, as standalone techniques [24] or combined with other magnetic or acoustic techniques. Inductive heating has been used to enable thermal imaging for defect detection in conjunction with eddy current testing [25,26].

Stress rehabilitation may employ stress annihilation for lowering localized stresses or quenching for increasing them. Stress annihilation can be achieved using localized heating methods [27], while localized stress strengthening may be achieved by localized heating and consequent quenching. Either way, localized heating is necessary for stress rehabilitation.

Having the motivation to provide technologies for stress distribution monitoring and rehabilitation of magnetic steels, three different methods for stress distribution monitoring, followed by stress annihilation have been employed. The stress distribution methods have been the rather classic permeability measurement [28], the non-linear acoustic methods based on the magnetostrictive delay line (MDL) principle [29], as well as the high-frequency eddy current based response [24]. In Section 2, the sensing means are presented in brief and their stress monitoring potential is demonstrated through indicative results on magnetic steels. Then, in Section 3, an induction heater is used to locally heat the corresponding volume suffering from stresses, followed by stress distribution monitoring, thus demonstrating an integrated method for stress annihilation.

## 2. Stress Monitoring

As mentioned in the previous section, stress monitoring has been carried out using three different methods for stress distribution monitoring: the localized and distributed monitoring of the permeability based on the B-H loop measurement, the MDL non-linear acoustic response for magnetoelastic and sound velocity non-uniformity, as well as the eddy current localized and distributed monitoring.

Without any loss of the generality, 1500 mm long, magnetic low carbon steel rods of 10 mm and 12 mm diameter were used. The actual monitored length was 500 mm, for practical experimental reasons. The proposed procedure is applicable on other types of steel rods, or pipelines (tubes), or orthogonal cross section magnetic steels.

**Figure 1:**
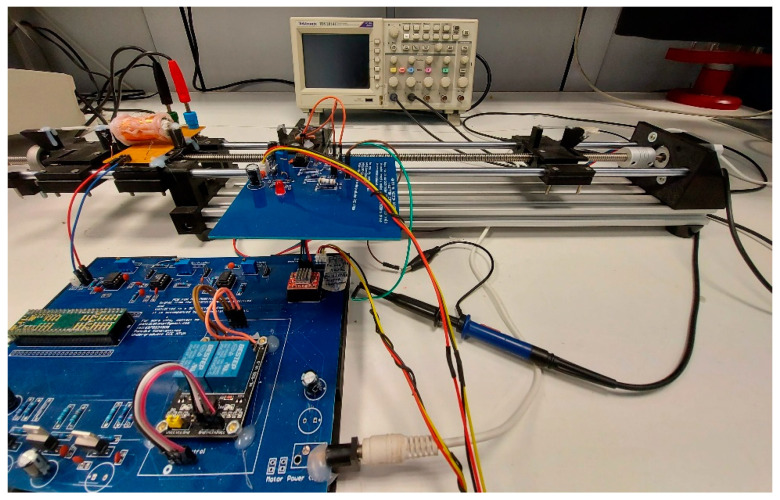
Linear translator of the three sensing heads.

All three measurement techniques (permeability, MDL, and eddy current measurements) used the same linear translator, as illustrated in Figure 1, to move the stress sensing means along the length of the rod. The motion of the linear translator was controlled by a microcontroller development board (Teensy 3.6), combined with a stepper motor driver (A4988), controlling a stepper motor. The uncertainty of the position of the sensing head was 0.1 mm, typical for such linear translators based on stepper motors.

Stresses were induced into the rods through controlled hammering: the rod was glued on a flat surface to secure its position and a 25 mm diameter steel sphere was dropped from a height of 1000 mm on two positions along the rod, at 35 mm and 70 mm from the starting measuring point, which was set at 100 mm from the one end of the rod.

In the following subsections, the results on the stress monitoring through magnetic, magnetoelastic, and eddy current measurements are presented. Three rods of 10 mm and 12 mm diameter have been tested, all with very similar responses. The results shown in this paper refer to the 10 mm rod. Error bars are in the order of 0.5–1% not shown on the figures.

### 2.1. Permeability Monitoring

It has been proven that the magnetic permeability tensor is monotonically dependent on the stress tensor, by comparing the actual localized strains of a given surface or volume of the material, and the corresponding residual stresses along different directions, with the amplitude of the localized surface or bulk permeability respectively along the same directions [30].

**Figure 2:**
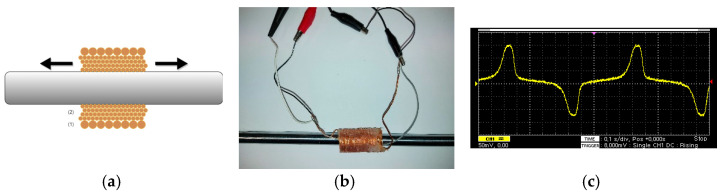
Experimental arrangement for B-H loop stress monitoring in rods: (**a**) the schematic of the primary (1) secondary (2) coil arrangement; (**b**) the actual set-up; (**c**) typical waveform of the secondary coil output.

Such a measurement can be realized by employing a primary-secondary coil arrangement, as schematically depicted in Figure 2a, and shown in Figure 2b. The secondary (search) coil was wound around an epoxy tube substrate manufactured by a 3D printer, while the primary coil was wound around and on top of the secondary coil, after setting a plastic insulator on top of the secondary coil. The secondary coil was made of high temperature resistance enameled copper wire of 0.1 mm diameter, having 300 turns in four layers. The primary coil was a high temperature resistance enameled copper wire of 0.5 mm diameter, having 100 turns. The length of the substrate was 60 mm and its internal and external diameter was 11 mm and 13 mm respectively.

Sinusoidal current of 0.1 Hz and amplitude of up to 1 A, was transmitted through the primary coil to magnetize the steel rod. The current waveform was generated by a Teensy microcontroller development board and amplified by a zero feedback current amplifier. The output of the secondary coil (a typical waveform is illustrated in Figure 2c) was driven to an operational amplifier controlled by the same microcontroller. Permeability measurements are carried out after minor-loop demagnetization: a 10 Hz sinusoidal excitation field is applied, starting at 10 A and decreasing down to 0 A, in steps of 0.1 A. The parameter being monitored is the peak of the output waveform which is proportional to the maximum differential magnetic permeability. The time required to obtain this parameter for one position along the rod is 10 s. Measurements of three and five periods have also been realized with no significant difference in the response.

Several research groups employ monitoring parameters obtained from the M-H loop, such as coercivity, which involve uncertainties due to the electronic or digital integration. The method presented in this paper employs permeability measurements which offer better sensitivity and direct proportionality to the residual stresses, as proven in previous works [14].

The length of the magnetic steel rod was long enough, offering negligible demagnetization factor, thus not requiring closing magnetic circuit. This is applicable in several industrial production and steel structures. Concerning industrial production of rods, the production is continuous or batch type of 6 m long, thus resulting in negligible demagnetization factor. In steel structures, the rod is practically attached to other magnetic steel structures, which allows not closing the magnetic circuit.

**Figure 3:**
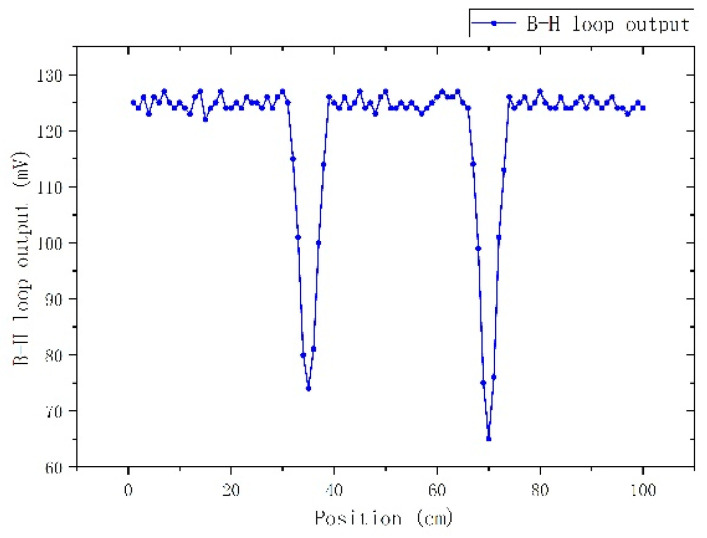
Dependence of the maximum differential permeability on the position of the primary-secondary coil, representing the effect of stress due to the hammering of the magnetic steel rod.

Following this experimental set-up, the magnetic permeability was monitored every 10 mm along the length of the magnetic steel rod. Indicative results are illustrated in Figure 3. The decrease of the output voltage, equivalent to the decrease of the magnetic permeability, is the actual indication of the residual stress at the vicinity of the rod, where the steel sphere dropped on. The actual amplitude of stress can be provided by comparing the permeability drop against the magnetic steel calibration curves (MASCs) [14]. From Figure 3, it can be seen that the effect of stress is observed along a length in the order of ±30 mm from the center of the hammering.

### 2.2. MDL Stress Monitoring

**Figure 4:**
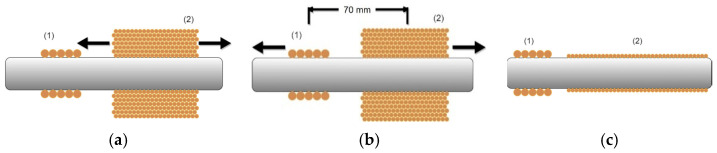
MDL set-ups aiming at stress distribution monitoring: (**a**) magnetoelastic uniformity measurement, where the excitation coil (1) remains in a fixed position and the search coil (2) moves along the length of the magnetic rod, with an output dependent on the localized residual stresses; (**b**) sound velocity measurement, where the excitation (1) and search (2) coils are fixed in distance and the whole assembly moves along the length of the rod, monitoring the change of the longitudinal sound velocity or the corresponding changes in the MDL delay time; (**c**) fast magnetoelastic uniformity tests indicating the difference in residual stresses between consequent infinitesimal volumes, where (1) is the excitation coil and (2) the long search coil.

Three different approaches have been implemented using the MDL technique, as depicted in Figure 4. All of them used the coil–coil arrangement, as described in [31,32]. According to this arrangement, an excitation coil of 10 turns in one single layer, made of 0.1 mm enameled copper wire, resulting in 1 mm long excitation coil, was set on top of a polymeric substrate made by 3D printing technology. The length of the substrate was 10 mm and its internal and external diameter was equal to 11 mm and 13 mm respectively. The excitation coil was used to transmit pulsed current of a peak up to 30 A, of 3 μs duration and period of 1 ms. The pulsed excitation current was controlled by a Teensy microcontroller development board and a zero feedback current amplifier. This current pulse was responsible for the generation of an elastic signal at the point of current excitation of the magnetic (and magnetostrictive) rod. Then, this elastic pulse was propagating as elastic wave along the two directions of the length of the magnetic rod and was detected by the search coil. The 300 turn search coil used the same substrate and was made of 0.05 mm enameled copper wire. Attention was paid to restrict the length of the search coil down to 2 mm in order to minimize the distortion of the detected propagating pulse. In all cases, the peak-to-peak amplitude of the voltage output of the search coil was the monitored magnitude.

According to the first arrangement (Figure 4a), the monitoring of the residual stresses is depicted as the dependence of the voltage output of the search coil on the position of the search coil. The excitation coil remains fixed at a given position while the search coil moves along the length of the magnetic rod, as described in [31], with a minimum distance from the excitation coil equal to 70 mm. Apart from the non-uniform magnetoelastic response due to the residual stresses, the MDL output suffers an exponential decay mainly attributed to the coupling between the excitation and search coil, and, to a lesser extent, to the acoustic attenuation of the rod. The transmitted current generates a magnetic field biasing the search coil. This biasing field depends linearly on the inverse distance between the excitation and search coils. As the dependence of the MDL output on the bias field is known [33], the response of the search coil was normalized with respect to the biasing field effect, and then the monitoring of residual stresses became apparent.

The second arrangement (Figure 4b) is based on the sound velocity measurement and monitoring, as depicted in [32]. Thus, the localized longitudinal sound velocity of the magnetic rod is the indication of the localized stresses in the volume between the excitation and search coils. According to this arrangement, the distance between the excitation and search coils is fixed at a distance of 70 mm, where the output signal is well separated from the induction impulse due to the excitation pulse. Then, the two-coil assembly is translated along the length of the magnetic rod and the sound velocity is monitored. A Teensy microcontroller development board has been used to measure the time delay between the excitation and the received signal from the search coil. This way, the time delay defines the longitudinal sound velocity in the above-mentioned volume. In mechanically harder rods, the time delay between the excitation and output pulses is expected to be shorter, in accordance with a higher sound velocity.

**Figure 5:**
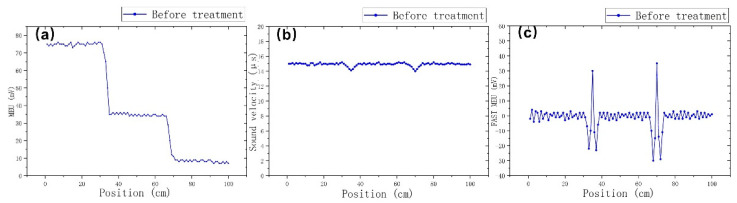
MDL response dependence on residual stresses: (**a**) MDL magnetoelastic uniformity (MEU); (**b**) sound velocity distribution; (**c**) fast magnetoelastic uniformity measurements.

The third arrangement is based on the set-up described in [34], the so-called fast magnetoelastic uniformity set-up. According to this set-up, shown in Figure 4c, the search coil is long and stationary, while the excitation coil is the one used in the previous two set-ups. For the needs of this set-up, a 1000 mm long plastic tube of 11 mm internal diameter and 15 mm external diameter was employed. A single turn solenoid of 0.05 mm enameled copper wire was wound along the whole length of the plastic tube. Transmitting pulsed current in the above-mentioned excitation coil, the elastic signal propagates and is detected in each infinitesimal part of the coil, corresponding to infinitesimal part of the tested rod. The residual stresses in the corresponding infinitesimal volumes of the magnetic rod determine the local voltage output. Thus, the level of the consequent voltages is the indication of the difference of residual stresses between two successive volumes in the magnetic rod. According to these three experimental set-ups, the MDL response is monitored for the same rod. Figure 5 illustrates the response of the three MDL set-ups: Figure 5a illustrates the stress dependence on the magnetoelastic uniformity response, Figure 5b the stress dependence on the longitudinal sound velocity and Figure 5c the stress dependence on the variation of consequent infinitesimal volumes of the magnetic rod.

According to the first set-up, the position of the excitation coil remains fixed while the search coil is moving along the length of the magnetostrictive delay line. Thus, the effect of the residual stresses is reflected on the dependence of the search voltage output on the distance between the excitation and search coil, accumulating the signal distortion. The second one maintains the distance between the two coils at 70 mm in order to have a fixed distance between the two coils, permitting the measurement of the longitudinal sound velocity in the volume of this 70 mm length of the rod: thus, moving the fixed distance assembly of the two coils allows for the monitoring of the changes in sound velocity along the length of the rod. The third type of measurement employs a long searching solenoid, permitting distribution monitoring of the gradient of residual stresses along the whole volume of the rod covered by the search coil. Each method has advantages and disadvantages: the first one is the simplest possible arrangement with a fast response with respect to the permeability measurement and the disadvantage of losing sensitivity due to the significant drop of the voltage output, if several volumes of induced stresses are involved. The second one offers a direct indication of stresses due to the change in the sound velocity, with the disadvantage of low spatial resolution, since each monitored volume of the rod involved lengths as large as 70 mm. The third one has the inherent advantage of fast gradient stress monitoring due to the precise reading of the whole volume of the rod covered by the search coil, with the disadvantage concerning the disability of monitoring the actual amount of localized residual stresses.

### 2.3. High Frequency Eddy Current Based Stress Monitoring

The high frequency arrangement follows the same set-up as the one illustrated in Figure 2. The only difference is the excitation frequency and current. The excitation frequency was fixed at 500 kHz, while the maximum current amplitude was limited to 100 mA. This type of measurement is able to mainly monitor geometrical changes of the surface of the rods and not residual stresses, since the high frequency is screening the microstructural effects, which occur at a much lower frequency inductive response.

**Figure 6:**
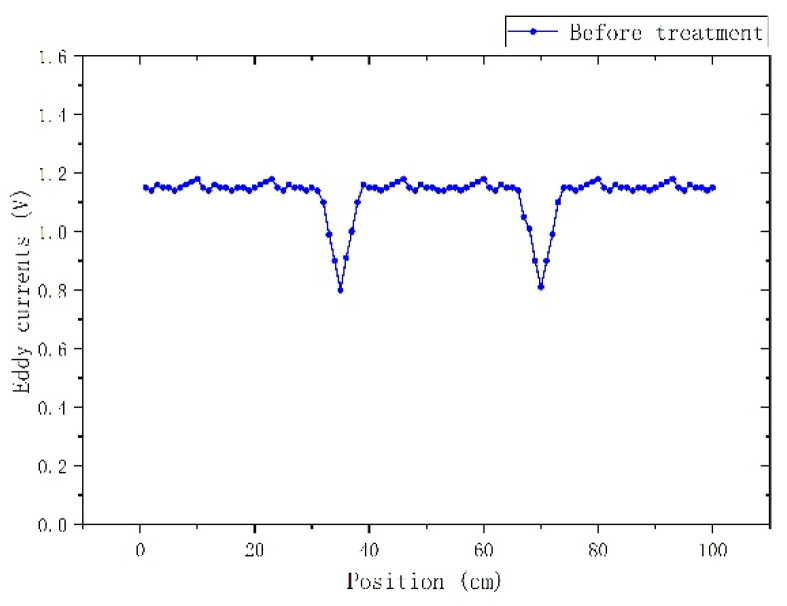
Eddy current monitoring of geometry changes.

According to this arrangement, stress and geometry changes along the length of the rod are depicted, as illustrated in Figure 6. At such high frequencies, the output depends on the skin depth, which is in turn dependent on the amplitude of permeability, which depends on the excitation field. The rest of the parameters (resistivity and excitation frequency) remain constant. It has been out of the scope of the paper to calculate the precise skin depth. For this reason, the excitation current frequency was set at 500 kHz. The maximum and minimum skin depths correspond to the minimum and maximum permeability of the material at 500 kHz. The minimum and maximum relative permeability of the rods has been determined to range from 10 and 1000, for the given amount of excitation current. Thus, assuming that the resistivity of the material is 400 μΩmm, for f = 500 kHz, the skin depth was calculated according to the typical skin depth equation $\delta =\sqrt{\frac{2\rho }{2\pi \omega \mu_{r}\mu_{ο}}}$, to be in the range between $\delta_{\min}=\sqrt{\frac{20\;\times \;10^{-6}}{10}}\;m≅1.5\mathrm{mm}$ and $\delta_{\max}≅\sqrt{\frac{20\;\times \;10^{-6}}{1000}}\;m≅0.15\;\mathrm{mm}$. Practically, the skin depth is below 1 mm, thus permitting measurements of geometrical surface non-uniformities.

## 3. Stress Annihilation

Stress rehabilitation has been reduced to stress annihilation for the needs of the experiment described in the current paper. Stress annihilation is based on localized induction heating.

**Figure 7:**
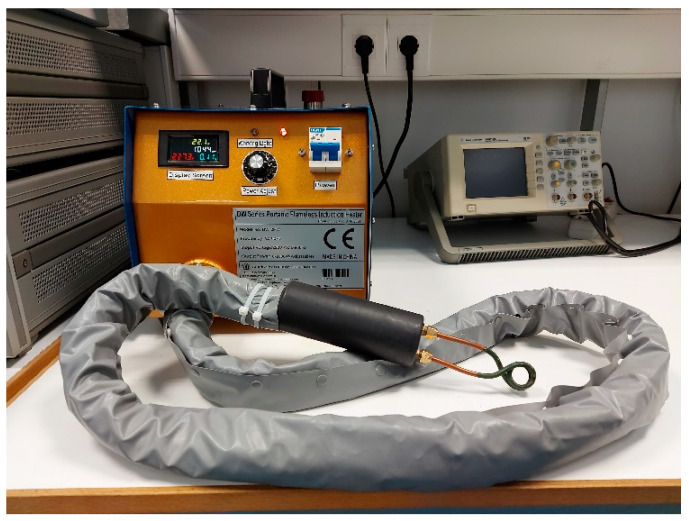
Induction heater.

The induction heater is the DW-2KW model of Guangzhou Durowelder Limited (Figure 7). A two-turn coil of copper tube, permitting water cooling is arranged around the rod, able to provide temperatures from RT up to the one third of the melting point of the rod, in other words up to 450 °C, considering 1350 °C as a typical melting point of the low carbon magnetic steels.

**Figure 8:**
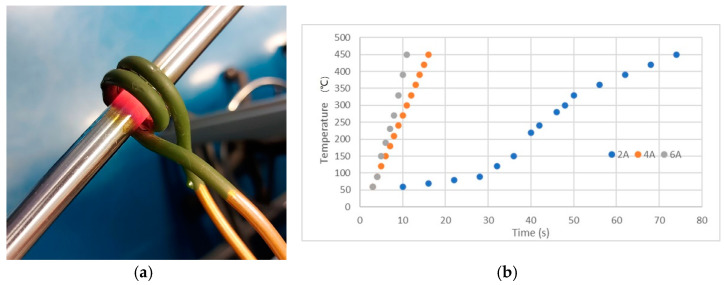
(**a**) Induction heating; (**b**) Temperature dependence on time, for 2A, 4A, and 6A.

The heating process has been calibrated using Pt100 temperature sensors. Figure 8a shows the coil of copper tube heating a rod. Typical temperature dependence on induction heating current time, for different current amplitudes (2A, 4A, and 6A), is illustrated in Figure 8b.

**Figure 9:**
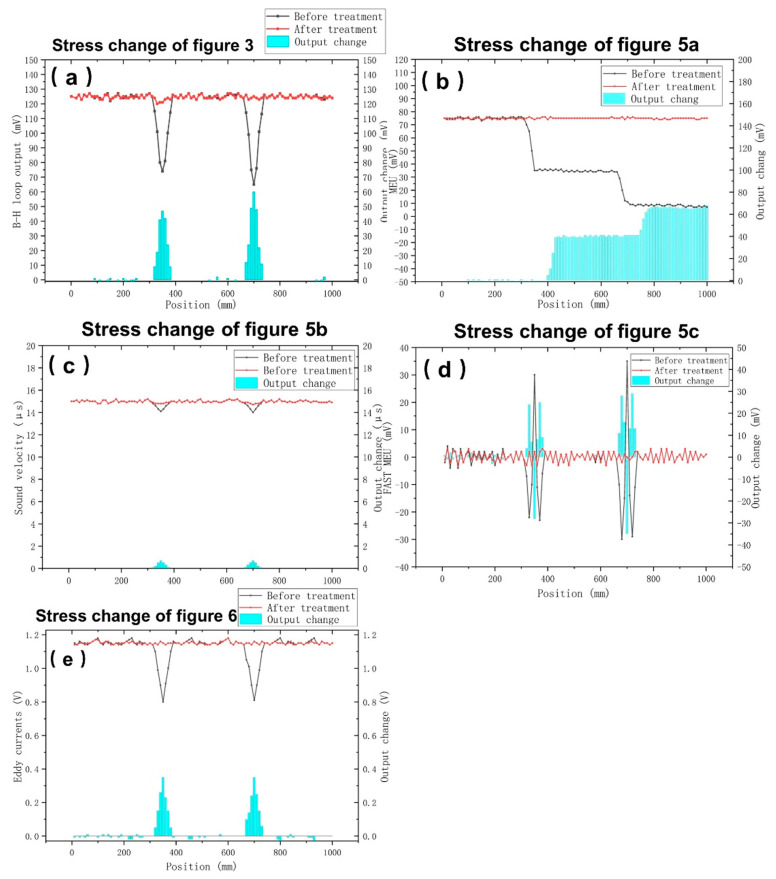
Change of the response of Figure 3, Figure 5a–c and Figure 6 showing as (**a**–**e**) in magnetic rods after 5 s localized induction heating.

Once the defective region was identified via any of stress monitoring methods described above, induction heating was applied for 5 s at the center of the region. Then, the stress monitoring was repeated using all methods. Figure 9 compares the response of all five types of measurements, before and after induction heating. No pre-tempering process was applied in our samples.

It was out of the purpose of this paper to calculate emissivity and related temperature effect on stress annihilation. This is currently under research, employing ANSYS for the finite element analysis. The Curie point of the rods is close to 600 °C, well below the maximum 450 °C temperature used in the described treatment. However, measurements were repeatable, even passing the Curie point and cooling down at room temperature with a cooling rate as low as 1–5 K/min, to avoid phase transformations. The post treatment monitoring of permeability or magnetostrictive delay line response took place 24 h after the heating process. The time needed to complete the process is considered as a disadvantage of the method in the case of stress annihilation certification. Future work is underway to estimate and project the expected room temperature permeability or the magnetostrictive delay line response based on measurements of relatively elevated temperatures, such as 100 °C or 200 °C, in order to shorten the time needed for the complete procedure.

## 4. Discussion

### 4.1. On the Stress Monitoring and Annihilation

From all these five different measurements presented in this paper, the following comments are derived:

- All measurements are in a relatively good agreement. Stresses are detected at the same positions, which is the important parameter to be detected. Bearing this in mind, the stress annihilation can be realized by localized heating, which, from the initial evidence and performance reported in this paper, has a promising potential.
- The permeability measurements can be used to determine the actual residual stresses. This comes at the cost of the speed of monitoring: at 0.1 Hz per point, at least 1000 s are required for 100 measurements. The spatial resolution of this measurement is in the order of 10 mm, due to the length of the search coil.
- The magnetoelastic uniformity indicates the consequent decrease of the output voltage, demonstrating an accumulation of stresses in the corresponding response. However, the method is fast, requiring a few tens of seconds for 100 measurements. Since the MDL voltage output is correlated with the permeability of the tested material [28], the correlation of permeability and residual localized stresses can also be determined. However, it was out of the scope of this paper to provide such detailed information. The variation of the voltage output suffices to decide whether rehabilitation through localized heating is necessary. Future work is underway to describe in detail the amount of current and time required, involving multi-parametric finite element analysis. The spatial resolution of this measurement is in the order of 1 mm, due to the length of the search coil.
- The sound velocity monitoring indicates the actual residual stresses. The speed of measurement is much higher than the permeability measurements, requiring a few tens of seconds to perform 100 measurements. These measurements are complementary to the magnetoelastic non-uniformity measurements, providing additional proof of residual stresses. However, the spatial resolution of the sound velocity measurement is in the order of 70 mm, due to the distance required between excitation and search coils.
- The fast magnetoelastic uniformity measurement offers the indication of the change of residual stresses at consequent infinitesimal point. It is the fastest method from all, requiring a few milliseconds to monitor the whole length tested, with the highest possible accuracy. Apart from being the fastest measurement from all methods studied in this paper, it also offers sufficiently good results, offering signals illustrating residual stresses at the same position such as permeability measurements, the other magnetoelastic measurements, as well as the eddy current measurements. The spatial resolution can easily be below 0.1 mm [32], dependent on the clock of the oscillator, performing the signal processing for the A/D conversion process.
- The eddy current measurement can determine localized geometrical changes, such as those caused by the steel sphere hammering. Such measurement is fast due to the high excitation frequency. Bearing in mind that the eddy current response depends on the product of conductivity and permeability, having determined the amplitude of permeability by low (and high) frequency measurements, the conductivity variation can also be determined, which may be useful for certain applications. The spatial resolution of this measurement is in the order of 1 mm, due to the length of the search coil.
- As a result of optimum instrumentation and measurement, as well as monitoring time, the fast magnetoelastic uniformity measurement based on the MDL technique and the eddy current geometrical changes measurement can be used for the precise and complementary determination of residual stress determination. However, if it is not possible to use the long coil method required for the fast magnetoelastic measurement, then the combination of the magnetoelastic uniformity and the eddy current measurement should also be acceptable, at the expense of time monitoring.

Table 1 summarizes all advantages and disadvantages of each method.

The uncertainty of measurement concerns the uncertainty of residual stress measurements using XRD Bragg–Brentano set-up and neutron diffraction for surface and bulk residual stress determination respectively, demonstrated in previous publications of our group [11]. Concerning the annihilation process, the results are rather promising. The reduction of residual stresses, as it is depicted in the five types of measurements is obvious and offers a rehabilitation larger than 90% in all cases.

### 4.2. SWOT Analysis

#### 4.2.1. Strengths of the Method

Ability to monitor non-uniformities and provide stress annihilation. Several applications, namely seamless tubes for heat exchangers, welds in shipyards and other areas, and testing of critical steel structures using rods or tubes. The effectiveness of the proposed methodology is the stress annihilation only in the volumes suffering large residual stresses, without affecting the robustness of the rest of the material.

#### 4.2.2. Weaknesses

**Figure 10:**
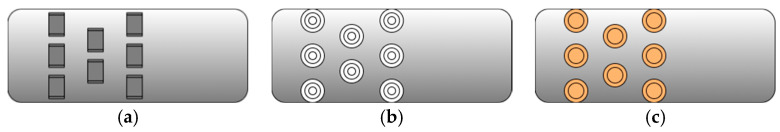
The proposed solution for point stress monitoring: (**a**) consequent rings of Hall or MR sensors with radial shift to cover the whole area; (**b**) pancake coils to excite and detect localized elastic pulses; (**c**) eddy current probes to monitor localized surface non-uniformities.

**Figure 11:**
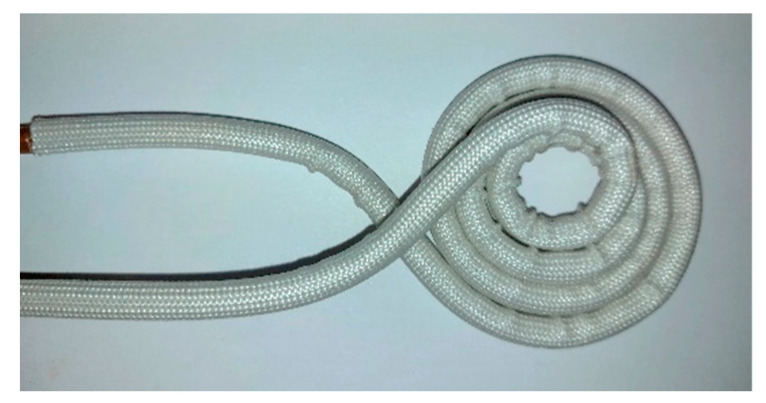
The proposed localized RF induction heater: pancake coils are used to cause localized heat.

Disability of localized heating within the volume under the sensing means. The averaging of stresses in the response of the three types of stress measurement and thermal annihilation may result in curing no-stress areas, resulting in softening of their mechanical properties. Thus, the presented method is limited to small diameter rods and tubes. The solution to monitor larger areas is peripheral stress monitoring, by using B-H loop techniques (or the MMM technique, using Hall or MR sensors in form of rings for flux leakage based stress monitoring), the MDL technology by rotating surface coils and the eddy current method by using localized eddy current probes. However, these monitoring techniques refer to surface stresses. Figure 10 depicts the proposal for such point stress monitoring techniques. Figure 10a illustrates the consequent rings of Hall or MR sensors, Figure 10b illustrates the localized pancake coils for magnetoacoustic monitoring, and Figure 10c illustrates the localized eddy current probes along the length of the rod or tube. The solution for corresponding stress annihilation is based on the use of small-scale pancake coils around the monitored stress, as depicted in Figure 11. Work is under way to develop these methods of stress measurement and annihilation.

#### 4.2.3. Opportunities

The technological opportunities based on the presented method are related to the disruptive application of the method in the three areas, mentioned above. In seamless tubes for heat exchangers, the final advantage is the certification of the level of stresses along the length of the tube, instead of a standard-based approach of stress level hypothesis. In welds, the monitoring of the localized stresses in the heat affected zone and the fusion zone is the actual proof of the performance of the weld, while the localized RF induction heating, followed by stress monitoring process is the actual certification of the stress relief caused by the welding. Testing critical steel structures of rods and pipelines becomes more effective than the classic crack monitoring. Apart from monitoring localized residual stresses, responsible for the initiation of localized nano-cracks, the proposed method can provide the localized stress annihilation and the final certification of using or not the given critical steel structures.

This way, the steel products in the form of rods is well certified, while the critical steel structures may also have a corresponding certification, declaring their lifetime.

#### 4.2.4. Threats

The possible threats of the proposed method for stress monitoring and annihilation can be the absence of national and international standards, allowing for the use of the method. Therefore, the submission of the proposed methodology to national or international standardization organizations in order to investigate and finally accept the method as the proper tool for stress monitoring and rehabilitation, possibly including other methods, like the ones described in this chapter, becomes mandatory. Furthermore, a faster response to such a threat is related to the development of internal procedures proving stress monitoring, or even magnetic permeability measurements or properties related to magnetic permeability, to provide the localized stresses and offer stress rehabilitation using RF induction heating.

The method can be used for other steel products and critical structures—namely plates, shafts, etc.—following the same methodology.

## 5. Conclusions

The proposed technology is good enough to provide localized stress monitoring and annihilation, improving the lifetime of the magnetic rods. The optimum measuring means are related to the sound velocity non-uniformity and the fast magnetoelastic uniformity provided by the MDL technique, as well as the geometrical inhomogeneity, provided by the eddy current measurements.

## Figures and Tables

**Table 1:** Advantages and disadvantages of the three or rather five different methods of measurement.

Sensor	Sensitivity of Measurement	Uncertainty of Measurement	Speed of Measurement	Ease of Measurement	Spatial Resolution
Permeability sensor	Able to detect 10 MPa residual stress	Certified <1%	10 s per point	Easy: small electromechanical coil–coil arrangement	10 mm
Magnetoelastic uniformity sensor	Able to detect 10 MPa residual stress	Assumed to be <1%	1 ms per point	Easy: small electromechanical coil–coil arrangement	1 mm
Sound velocity uniformity sensor	Able to detect 10 MPa residual stress	Assumed to be <1%	1 ms per point	Easy: small electromechanical coil–coil arrangement	70 mm
Fast magnetoelastic uniformity sensor	Able to detect 10 MPa residual stress	Assumed to be <1%	1 ms per 1000 points (integrated measurement)	Not easy: long search coil	0.1 mm
Eddy current sensor	Not applicable	Not applicable	1 ms per point	Easy: small electromechanical coil–coil arrangement	1 mm
